# Changes in Quercetin Derivatives and Antioxidant Activity in Marigold Petals (*Tagetes patula* L.) Induced by Ultraviolet-B Irradiation and Methyl Jasmonate

**DOI:** 10.3390/plants11212947

**Published:** 2022-11-01

**Authors:** Ji Hye Kim, Shucheng Duan, You Jin Lim, Seok Hyun Eom

**Affiliations:** Department of Smart Farm Science, College of Life Sciences, Kyung Hee University, Yongin 17104, Korea

**Keywords:** flavonoid, growth regulator, marigold flower, quercetin-3-*O*-glucoside, quercetin-7-*O*-glucoside, ultraviolet B

## Abstract

Marigold petals contain numerous antioxidative flavonoids and carotenoids that can be affected by environmental stressors. There is yet no detailed study on the relationship between phytochemical accumulation and stressors in marigold petals. This study evaluated quercetin derivatives and antioxidant activity in marigold petals in response to ultraviolet-B (UV-B) irradiation and methyl jasmonate (MeJA) treatment. The limiting UV-B radiation intensity and MeJA dose that caused no wilting damage under 1-h daily treatment for 10 days were <2 W∙m^−2^∙s^−1^ and <10 mM, respectively. Marigold petals contained three major flavonoids, quercetin-7-*O*-glucoside (Q7G, 6.6 mg∙g^−1^dw), quercetin-3-*O*-glucoside (Q3G, 62.7 mg), and quercetin (26.6 mg), possessing different antioxidant potential and exhibiting the highest power in quercetin and next value in Q7G. Single UV-B irradiation exerted a limited effect on the changes in the content of the three quercetin derivatives, whereas combined treatment with 1 W UV-B radiation and 5 mM MeJA resulted in the highest total quercetin content, showing >20% increase compared to that without treatment. This increase primarily resulted in an increase in quercetin content. MeJA treatment positively affected the increase in Q3G and Q7G contents in a dose-dependent manner during the 10-d experimental period but exerted no considerable effect on quercetin accumulation. The antioxidant activity was increased when flowers were exposed to mild MeJA treatment of 5–10 mM. UV-B irradiation decreased the antioxidant activity of marigold petals, but this decrease could be compensated by MeJA treatment.

## 1. Introduction

The French marigold (*Tagetes patula* L.), belonging to the *Asteraceae* family, has been cultivated throughout the world [1]. Traditionally, it is grown as an ornamental plant in gardens because of its long flowering period and attractive bright yellow flowers. Recently, it has been recognized as an excellent food additive for health-related effects [2,3]. This is primarily because of its numerous secondary metabolites that might possess pharmacological properties, such as antioxidant, anti-inflammatory, and antifungal activities [4,5,6]. Among those metabolites, flavonoids play a more important role in the pharmacological activity of flowers, and representative compounds such as quercetin are used as indicators to evaluate the quality of raw materials [7,8].

Flavonoids play an essential role in mediating plant responses to biotic and abiotic environmental factors. For instance, it protects plants from biological stressors such as pests and abiotic environmental stressors, including UV light absorption. In particular, abiotic stress affects phytochemical accumulation in plants because stressful environments activate endogenous defense mechanisms in plants, causing the activation of metabolites or accumulation of secondary metabolites such as carotenoids and flavonoids [9,10]. Nevertheless, there is still no clear information on the effects of abiotic stress on marigold flowers, especially the biosynthetic regulation of secondary metabolites.

Recently, methods for artificially treating stress have been devised to promote the accumulation of secondary metabolites such as flavonoids. UV light, an abiotic stress, increases the accumulation of UV-absorbing and defense-related phytochemicals, which is caused by the action of DNA photolytic enzymes and antioxidant systems and the expression of genes involved in UV protection and repair [11,12,13]. It was confirmed that flavonoids such as kaempferol and quercetin in broccoli were accumulated 24 h after UV-B treatment [14]. The total carotenoid content of postharvest chrysanthemum (*Asteraceae* family) was significantly increased after irradiation with UV-B [15]. It was reported that UV-B irradiation is a good strategy to increase the content of both total flavonoids and carotenoids in medicinal chrysanthemum [16].

Exogenous growth regulators, such as MeJA, have also been considered an excellent abiotic stress-inducing strategy to accelerate the accumulation of secondary metabolites [17,18]. Numerous studies have reported phytochemical content changes in various plants treated with MeJA [19,20]. For instance, thyme species treated with MeJA exhibited higher total phenolic and flavonoid contents than non-treated plants [21]. MeJA treatment has also been reported to improve flavonoid production in safflower (*Carthamus tinctorius* L.) by increasing the expression of genes related to flavonoid biosynthesis [22].

Previous research has reported on the increase in flavonoid content and associated gene expression by UV-B and MeJA treatments [23,24,25]. These compounds are produced as antioxidants for the removal of free radicals caused by abiotic stress. In apples, UV-B and MeJA treatments caused a significant increase in the content of anthocyanin and quercetin glycosides, along with a significant increase in the expression of related genes [23,24]. The application of artificial stress treatments in several plant species is a commonly used method for enhancing the production of phytochemicals [23,26,27]. However, there is no information on the effect of abiotic stress application on marigold flowers, although several studies have examined the phytochemical changes in marigold flowers during thermal processing or extraction procedure [28,29]. Exploring abiotic stresses such as UV-B irradiation and MeJA treatment may be a useful strategy to produce marigold flowers possessing higher contents of phytochemicals.

Considering information mentioned above, this study was conducted to (1) determine the conditions of UV-B intensity, MeJA concentration, and treatment duration that produce high flavonoid content, (2) quantitatively categorize quercetin derivatives in response to abiotic stressors, and (3) evaluate the relationship between stress-induced marigold flower extracts and their antioxidant activity.

## 2. Results

### 2.1. Morphology of Marigold Flowers Treated by UV-B Intensity and MeJA Concentration

**Figure 1:**
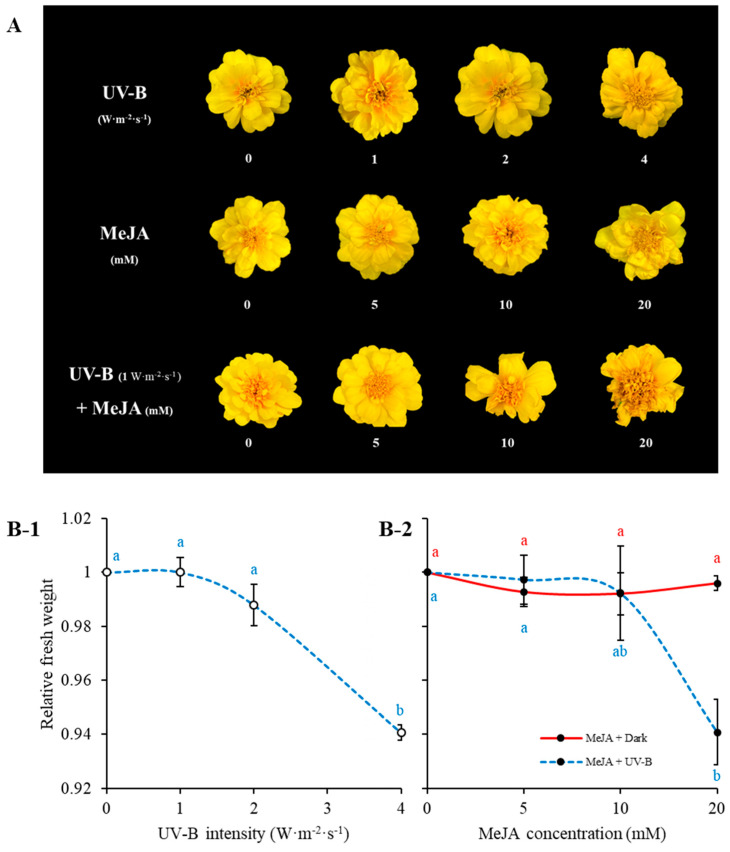
Morphological appearance of marigold flowers treated with different UV-B intensities and MeJA concentrations for 10 days (**A**). Each row in (**A**) represents treatment with UV-B alone (without MeJA), MeJA alone (without UV-B), and treatment by MeJA concentration under UV-B 1 W·m^−2^·s^−1^. Relative fresh weight of marigold flowers depending on UV-B intensities (**B-1**) and MeJA concentrations (with or without UV-B 1 W·m^−2^·s^−1^) (**B-2**) treatment for 10 h within a day. The relative weight was expressed as the mean ± SE value of the weight of triplicate samples. Alphabetically small and different letters on graphs indicates significant differences in Tukey’s studentized test at *p* < 0.05.

**Figure 2:**
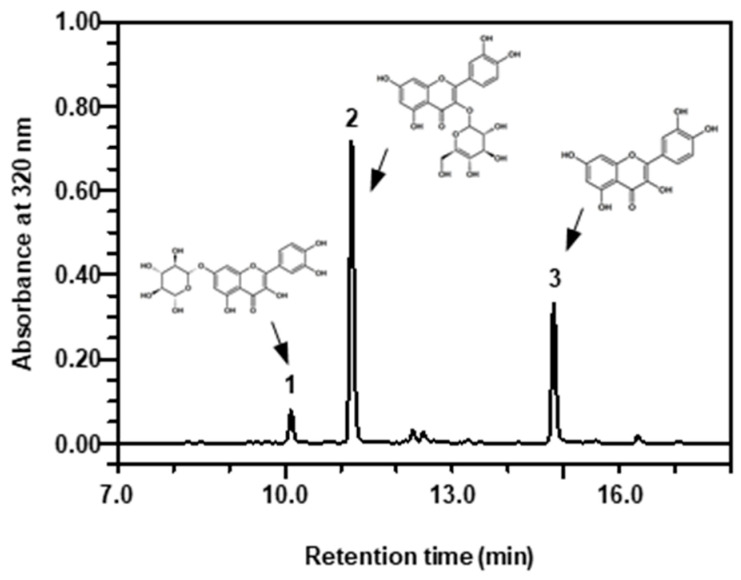
HPLC chromatogram of quercetin derivatives in marigold petals; 1, Quercetin-7-*O*-glucoside (Q7G); 2, Quercetin-3-*O*-glucoside (Q3G); 3, Quercetin.

Figure 1 shows the morphology of marigold flowers treated with UV-B radiation and MeJA for 10 days. High intensity (4 W·m^−2^·s^−1^) of UV-B irradiation induced severe wilting of flower petals, whereas low intensity (1 and 2 W·m^−2^·s^−1^) did not (Figure 1A). The fresh weight loss of petals measured under treatment with high intensity of UV-B is depicted in Figure 1B-1. MeJA treatment changed the color of petals from light yellow to heavy yellow compared to that without MeJA treatment. With increases in the concentration of MeJA, there was an increase in the severity of wilting symptoms (Figure 1A,B-2). Shrunken petal shape was frequently observed at MeJA concentrations of >10 mM (Figure 1A). Combined treatment of 1 W·m^−2^·s^−1^ UV-B and MeJA caused extreme wilting of marigold petals. The petals were severely dehydrated with UV-B irradiation combined with 20 mM MeJA, showing approximately 10% reduction in fresh weight compared with the control (Figure 1A,B-2).

### 2.2. Changes in Quercetin Derivatives in Response to UV-B Irradiation with or without MeJA Treatment

**Figure 3:**
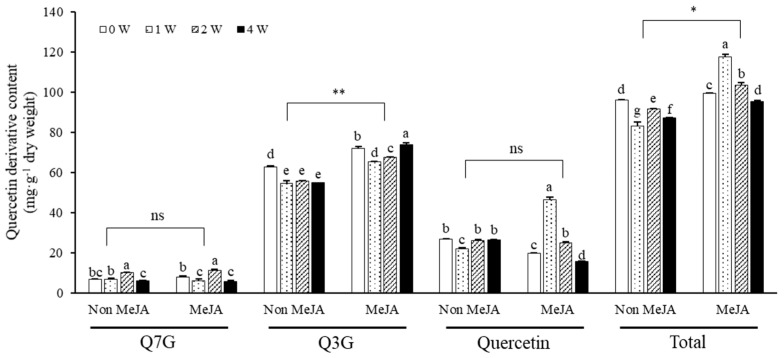
Quercetin derivatives content in marigold petals treated with MeJA (5 mM) or non-treatment depending on UV-B intensity (0, 1, 2, 4 W∙m^−2^∙s^−1^) for 5 days. All data is exhibited using mean value ± standard deviation within three replications (*n* = 3). Different letters within bar graphs of each group (Q7G, Q3G, Quercetin, and Total) indicate significant differences by Tukey’s studentized test (HSD) at *p* < 0.05. Significant difference between non-MeJA and MeJA in each group was performed by Student’s *t*-test. ns, *, and ** indicate statistically non significance, significance at *p* < 0.05, and significance at *p* < 0.01, respectively.

Three quercetin derivatives in marigold petals were detected on HPLC, exhibiting Q7G peak at 10.1 min on retention time, Q3G peak at 11.2 min, and quercetin peak at 14.8 min (Figure 2). Figure 3 shows the contents of quercetin derivatives in the petals of marigold flowers treated with 5 mM MeJA or without MeJA treatment under exposure to different UV-B radiation intensities of 0–4 W∙m^−2^∙s^−1^ for 1 h daily for 5 days. The marigold petals used in this experiment generally contained approximately 96 mg∙g^−1^ dry weight of quercetin derivatives. Among the experimental treatments, 1 W UV-B treatment with MeJA induced significant increases in the total quercetin derivative content, showing 117 mg g^−1^ dry weight. MeJA treatment exerted a positive effect of increasing the total quercetin derivative content, especially influencing UV-B irradiation where the content was the highest at 1 W UV-B irradiation and gradually decreased under increasing intensities. These quantitative changes in response to MeJA and UV-B treatments were primarily dependent on Q3G and quercetin contents. The increase in the total quercetin derivative content primarily resulted in the difference between non-MeJA and MeJA treatments for Q3G and between non-MeJA (approximately 25 mg of quercetin) and MeJA treatments under 1 W UV-B irradiation (approximately 46 mg of quercetin) for quercetin. Although the Q7G content showed no significant difference between MeJA and non-MeJA treatments, it was significantly increased under higher UV-B intensity, showing 10–11 mg∙g^−1^ dry weight. Q3G was a dominant quercetin derivative in the marigold petals, comprising approximately 55–72 mg∙g^−1^ dry weight. The petals contained approximately 20–46 mg of quercetin and approximately 6–11 mg of Q7G in single treatment of MeJA and UV-B. Q3G maintained a relatively high amount under non-UV-B treatment, but its content was decreased under UV-B exposure. Its content was higher under 5 mM MeJA treatment than under non-MeJA treatment. Under MeJA treatment, the decreasing pattern of Q3G content after UV-B irradiation was less than that under non-MeJA treatment. The quercetin content showed no significant patterns in response to single UV-B treatment; however, under MeJA treatment, its content increased and decreased according to UV-B irradiation intensity.

### 2.3. Short- and Long-Term Effects of MeJA and UV-B Treatments on Quercetin Derivatives

**Figure 4:**
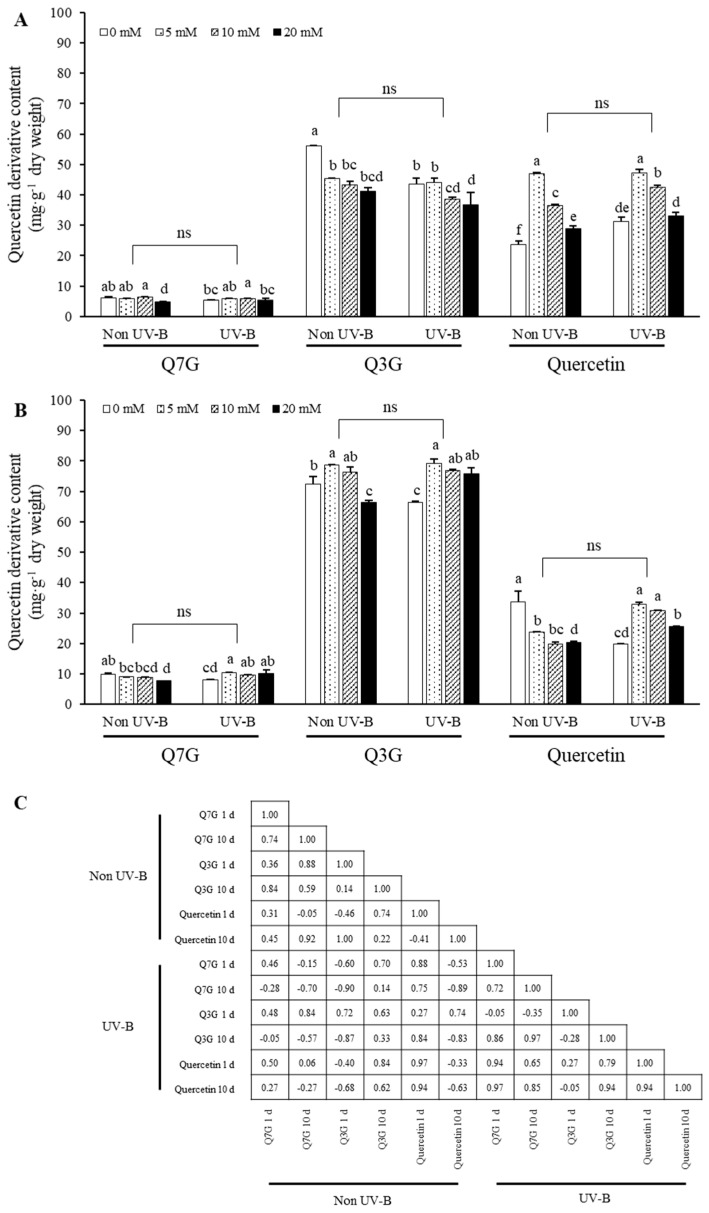
Quercetin derivatives content in marigold petals treated with different MeJA concentrations with/without UV-B (1 W∙m^−2^∙s^−1^) for 1 h/day × 1 day (short period treatment) (**A**) and 1 h/day × 10 days (long period treatment) (**B**). Correlation of individual flavonoids by treatment (**C**). Different letters within each group (Q7G, Q3G, and Quercetin) in (**A**,**B**) indicate significant differences by Tukey’s studentized test at *p* < 0.05. Significant difference between non-MeJA and MeJA in each group was performed by Student’s *t*-test. Correlation was performed with Pearson correlation coefficient test. Q7G, Quercetin-7-*O*-glucoside; Q3G, Quercetin-3-*O*-glucoside. Content of Q7G is expressed mg Q3G equivalents·g^−1^ dry weight. All data is expressed mean ± standard deviation of three replications.

The changes in quercetin derivative contents in either 1- or 10-day-treated marigold petals according to 0, 5, 10, and 20 mM MeJA concentrations under 1 W·m^−2^·s^−1^ UV-B treatment are shown in Figure 4. Marigold petals without MeJA and UV-B treatments showed increased total contents of quercetin derivatives when the treatments were applied for a longer period. However, the petals without MeJA treatment but exposed to UV-B radiation showed increased Q7G and Q3G contents but a decreased quercetin content.

The effect of MeJA treatment on quercetin content changes was higher during the early flowering period. In the 1-day treatment, the effect of UV-B irradiation was not distinct, showing similar patterns of quercetin content. However, the contents showed distinct differences among MeJA concentrations. A relatively low MeJA concentration (5 mM) significantly increased the quercetin content by approximately 2-fold compared to that without MeJA treatment. A higher MeJA concentration tended to reduce quercetin accumulation, exhibiting gradually decreasing patterns (Figure 4A). In the 10-day treatment, the quercetin content under different MeJA concentrations showed different patterns between non-UV-B and UV-B treatments. Under non-UV-B condition, the quercetin content was the maximum without MeJA treatment but decreased with MeJA treatment. However, under UV-B condition, the quercetin content was the minimum without MeJA treatment but significantly increased with MeJA treatment, exhibiting a similar pattern to that in the 1-day treatment. Regarding Q3G, MeJA treatment increased its content with longer treatment periods (10 days). MeJA treatment and UV-B irradiation decreased the Q3G content at 1 day. However, the effect of MeJA treatment differed according to the concentration. Lower MeJA concentrations of 5 and 10 mM induced an increase in Q3G content under non-UV-B treatment, whereas 20 mM MeJA treatment significantly decreased the content. This decrease at the high MeJA concentration was not observed under UV-B irradiation. Regarding Q7G, MeJA treatment exerted a negative effect on the increase in its content under non-UV-B condition. The Q7G content was significantly decreased at the highest MeJA concentration (20 mM). This negative effect of MeJA treatment on Q7G accumulation was not observed under UV-B irradiation; instead, it exerted a positive effect when the UV-B treatment period was longer (10 days). However, this positive effect was not dependent on the dose.

The correlation between each treatment group on days 1 and 10 was maximum in terms of quercetin content under UV-B irradiation (*r* = 0.94), and a moderate correlation was also found under non-UV-B treatment for Q7G content (*r* = 0.74). No correlation was found between the other treatment groups. The correlation according to the presence or absence of UV-B irradiation in each treatment group was high in the order of quercetin on day 1 and Q3G on day 1 (*r* = 0.97 and 0.72, respectively). There was no correlation according to UV-B treatment for other compounds. Quercetin and Q3G, which showed a high correlation, showed similar responses in their content according to MeJA concentration, irrespective of UV-B irradiation.

### 2.4. Antioxidant Activities in Response to UV-B Irradiation and MeJA Treatment Alone or in Combination

**Figure 5:**
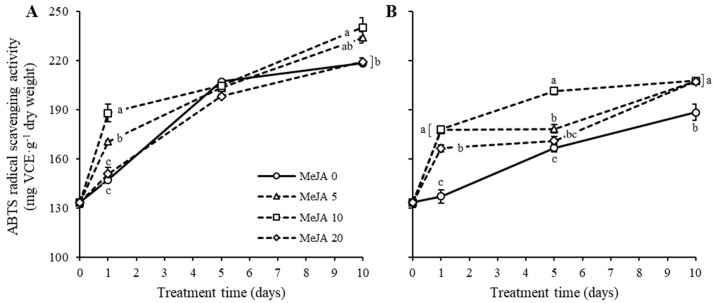
ABTS radical scavenging activity of marigold petals treated to series of MeJA concentration either without UV-B (**A**) or with 1 W∙m^−2^∙s^−1^ UV-B (**B**) for 10 days. Antioxidant activities were expressed as mg VCE·g^−1^ dry weight. VCE indicates vitamin C equivalents. Error bars on each graph indicates standard errors of triplicate samples. Statistical significance within treatment day was performed with Tukey’s studentized test (HSD) at *p* < 0.05.

Figure 5 shows the patterns of antioxidant activity of marigold petals treated with UV-B irradiation and MeJA for 10 days. The marigold petal extract demonstrated increasing antioxidant activities until day 10, showing approximately 133 mg VCE∙g^−1^ dry weight (mg VCE∙g^−1^ distilled water (DW)) on the first day and approximately 210–240 mg VCE∙g^−1^ DW on day 10 with MeJA treatment without UV-B irradiation (Figure 5A). The antioxidant activity was drastically increased at day 1, but the increasing pattern was slower between days 5 and 10 than that during the early period (Figure 5A). Under day 1 treatment with MeJA, the antioxidant activity in the extract was significantly high at 10 mM MeJA, whereas it was low at 20 mM MeJA, with levels similar to those without MeJA treatment. The antioxidant activity patterns among MeJA treatments did not significantly differ at day 5 but significantly differed at day 10 with similar patterns as those at day 1. In contrast, the antioxidant activities were relatively low under UV-B irradiation compared to those under non-UV-B irradiation (Figure 5A,B). Under UV-B irradiation, the marigold petals showed relatively low antioxidant activity increase with non-MeJA treatment, still showing an increasing pattern when the treatment period was longer. MeJA treatment exerted a synergetic effect on the increase of antioxidant activity under UV-B irradiation. The efficient concentration of MeJA was 10 mM that resulted in increased antioxidant activity within 5 days of treatment. However, the effect of concentration was not different at day 10, where a higher antioxidant activity (approximately 220 mg VCE g^−1^ DW) was maintained than that (approximately 180 mg VEC∙g^−1^ DW) under non-MeJA treatment (Figure 5B). Overall, the antioxidant activity of marigold petals was the highest at 10 mM MeJA without UV-B irradiation and a longer treatment period.

### 2.5. Correlation between Antioxidant Activity and Antioxidants

**Figure 6:**
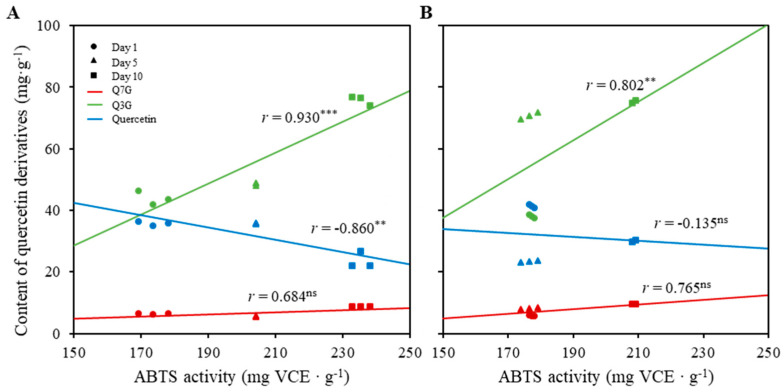
Correlation between ABTS radical scavenging activity and content of quercetin derivatives in 10 mM MeJA treatment without UV-B (**A**) and with 1 W∙m^−2^∙s^−1^ UV-B (**B**). ns, **, and *** indicate no significance, significance at *p* < 0.01, and significance at *p* < 0.001, respectively, in Pearson correlation coefficient test.

Figure 6 shows the correlation between ABTS radical scavenging activity and quercetin derivative content of marigold petals treated with 10 mM MeJA and 1 W∙m^−2^∙s^−1^ UV-B alone or in combination. The conditions of MeJA concentration and UV-B intensity had shown highest antioxidant activity among the experimental treatments (Figure 5). The ABTS radical scavenging activity correlated differently with each compound depending on the treatment period. In the absence of UV-B irradiation, the ABTS radical scavenging activities according to the treatment period showed a high positive correlation with the content changes of Q3G (*r* = 0.930), with strong significance among treatment periods (*p* < 0.001) (Figure 6A). However, the activities showed a negative correlation with the content changes of quercetin (*r* = −0.860), with strong significance among treatment periods (*p* < 0.01) (Figure 6A). Interestingly, the increases in ABTS radical scavenging activity along with treatment period were not dependent on the changes in Q7G content, with no significance between the content changes of Q7G and the activity changes of ABTS. In the presence of UV-B irradiation, the correlation patterns between the content changes of quercetin derivatives and activity changes of ABTS were similar in directional tendency. However, the correlations were different in the absence of UV-B irradiation, showing different patterns with treatment periods. The ABTS radical scavenging activities were similar on day 1 and day 5 treatments (Figure 6A), whereas they gradually increased in the absence of UV-B irradiation with an increase in treatment period (Figure 6B).

## 3. Discussion

Previous studies have reported that the application of UV-B radiation and the exogenous growth regulator MeJA can change the morphology and physiology of plants [23,24]. This phenomenon was also observed in our study, which showed the changes in wilting and fresh weight in different flowering stages of marigold petals. Interestingly, single MeJA treatment exerted a beneficial effect of maintaining the fresh weight of marigold petals (Figure 1). The reduction in fresh weight by UV-B treatment was found to be consistent with other studies as a consequence of the dependence on the expense of biosynthesis of flavonoids and related phenolic compounds [30]. However, several studies have reported that moderate treatment with UV-B radiation and MeJA in radishes, *Arabidopsis thaliana*, and faba beans can increase plant biomass [31,32,33]. These variations in morphological changes in response to a stressor can depend on the plant species or the type of stressor.

Single UV-B irradiation in marigold petals exerted no effect on increasing the total content of quercetin derivatives, but the content increased when it was combined with MeJA treatment (Figure 3). A decreasing pattern after the initial increase was found with an increase in UV-B radiation intensity, with the highest amount of quercetin derivative detected at 1 W·m^−2^·s^−1^ UV-B. These results suggest that a relatively low intensity is appropriate for quercetin derivative accumulation. The increase in the total quercetin derivative content was contributed by the increase in the content of the quercetin aglycone (Figure 3). The increase in aglycone content by moderate UV-B irradiation was similarly observed in both faba sprouts and roots [33].

In addition to the UV-B radiation intensity, MeJA concentration and treatment period affected the pattern of flavonoid accumulation. The content of quercetin glycosides varied with the progression of marigold flowers during 10 days (Figure 4). Irrespective of UV-B irradiation, a higher MeJA concentration exerted a negative effect on the accumulation of flavonoid content, suggesting that high MeJA concentrations induce excessive stress or exhibit toxic effects [34]. Based on these observations, we suggest that a MeJA concentration of ≤10 mM is effective for the accumulation of flavonoids in marigold petals. These data are consistent with those reported by Kianersi, describing that high MeJA concentrations increased the total flavonoid and total phenolic contents of lemon balm (*Melissa officinalis* L.) and the transcript levels of related genes [35]. Furthermore, the selection of the treatment period according to the purpose is considered to be important because the content of each compound varies according to the treatment period of UV-B irradiation and MeJA. These results indicate that the determination of MeJA concentration and treatment period considering the type and organ of the crop is a vital factor.

As mentioned earlier, both flavonoid content and antioxidant activity were increased when marigold flowers progressed (Figure 4 and Figure 5). Single UV-B radiation treatment exerted a negative effect on ABTS radical scavenging activity, but single MeJA treatment exerted a positive effect. The combination of UV-B irradiation and MeJA treatment alleviated the negative effect exerted by single UV-B radiation treatment. Moreover, the maximum correlation (without UV-B, *r* = 0.930; with UV-B, *r* = 0.802) was found between the antioxidant activity and flavonoid content for Q3G rather than quercetin, although it has been reported to exhibit a higher antioxidant activity than the quercetin glycoside [36,37,38]. This can be explained by the relative content differences among each compound.

An increase in quercetin content and quercetin glycoside content was observed in the early stage and later of stress, respectively. The different reactions between quercetin and its glycosides under stress factors might be related to the activation levels of antioxidant mechanism. These results suggest an increase in quercetin content for rapid ROS elimination in the early stage of stress. Moreover, during the later treatment period (day 10), increases in both aglycone and glycoside contents result in higher levels of hydroxylation (dihydroxy β-ring substitution forms such as quercetin 3-*O* and quercetin 7-*O*-glycoside) that act as antioxidants for ROS scavenging. This is presumed to be due to the induction of flavonoid synthesis [39,40,41,42].

## 4. Materials and Methods

### 4.1. Chemicals and Plant Materials

All solvents used in the analysis, including methanol and acetonitrile, were of HPLC analytical grade (Daejung Chemical & Metals Co., Ltd., Shiheung, Korea). Quercetin and quercetin 3-*O*-glucoside (Q3G) were purchased from ChemFaces Biochemical CO., Ltd. (Wuhan, China) as analytical standards. MeJA was purchased from Sigma-Aldrich (St. Louis, MO, USA).

Marigold seeds (Durango yellow) purchased from Asia Seed Co., Ltd. (Seoul, Korea) were planted in pots filled with a commercial horticultural soil (Baroker, Seoulbio Co., Eumseong, Korea). The seedling plants were grown under glass transparent natural light in a greenhouse at Kyung Hee University during the late spring season (Youngin, Korea) until the shoot length reached >15 cm. Among them, plants containing three or more flowers were selected and used in the abiotic stress experiment. The sample of each treatment was collected from more than 15 individual plants. The sample collections were performed three times (*n* = 3). Experimental treatment of stress inducers was initiated when the flowers were under freshly and fully blooming stage.

### 4.2. Treatment with UV-B Irradiation with or without MeJA for 5 Days

Five individual plants per treatment were set under the following conditions: DW or 5 mM MeJA treatment under 0, 1, and 2 W∙m^−2^∙s^−1^ UV-B. UV-B light used was a Narrowband TL 20W/01-RS Ultraviolet-B (Philips Co., Hamburg, Germany). To maintain the same conditions of MeJA-treated and untreated plants, the same amount of DW was sprayed as that for untreated plants. Both MeJA and DW treatments were prepared by adding 0.01% tween 20. Each plant was partially covered with a transparent plastic bag to prevent rapid evaporation of MeJA and DW immediately after spraying. After spraying and covering, the plants were irradiated with UV-B for 1 h per day (13:00–14:00 p.m.) for 5 days. Plants were kept in the greenhouse during the time, except during UV-B irradiation. UV-B irradiation was conducted in a facilitated plant growth room set with UV-B light, 23–25 °C, and 70–80% relative humidity. Flower petals were harvested after 5 day treatment and lyophilized (Freeze drier, Ilshin Biobase Co., Seoul, Korea). The dried petals were coarsely pulverized using a commercial home grinder and stored in a −20 °C freezer.

### 4.3. Treatment with MeJA under 1 W∙m^−2^∙s^−1^ UV-B Irradiation

MeJA solutions of 0, 5, 10, and 20 mM were respectively sprayed on the whole marigold plants. As mentioned in Section 4.2, MeJA and DW treatments were prepared by adding 0.01% tween 20. After spraying, the plants were partially covered with a transparent bag to prevent rapid evaporation. Plants treated with DW or MeJA were daily moved into a room facilitated with 1 W·m^−2^·s^−1^ UV-B for 1-h irradiation at every 13:00–14:00 p.m. for 10 days. After UV-B irradiation, the plants were moved back to the greenhouse. The petals of treated flowers were harvested on 1, 5, and 10 days after treatment. After freeze-drying, the sample was pulverized using a commercial grinder and used for further analysis.

### 4.4. Determination of Flavonoids by Reverse-Phase HPLC

The ground sample (20 mg) was extracted with 4 mL of a mixture solution containing 28:70:2 (*v*/*v*) of water, methanol, and formic acid, respectively. The extraction was performed in a shaking incubator for 12 h after sonication for 1 h and filtered using a 0.45-μm PTFE syringe filter (Futecs Co., Ltd., Daejeon, Korea). The extract was directly stored at 4 °C after the filtration and used to determine flavonoid content by HPLC analysis and antioxidant activity using ABTS radical scavenging activity. In the HPLC analysis, the solution of mobile phase consisted of water with 0.1% formic acid (mobile phase A) and acetonitrile with 0.1% formic acid (mobile phase B). The gradient program of the mobile phase was set as follows: 1–30% B for 0–10 min, 30–90% B for 10–23 min, 90% B for 23–25 min, 90%–1% B for 25–27 min, and 1% B for 27–30 min. The flow rate was maintained at 1 mL per min, and the injection volume was 20 μL. Peaks were detected at 350 nm using a Waters 996 photodiode array detector (Waters Inc., Milford, MA, USA).

### 4.5. Measurement of Antioxidant Activities

The antioxidant activity was determined by measuring the 2,2′-azino-bis (3-ethylbenzothiazoline-6-sulfonic acid) (ABTS) radical scavenging activity as described by Lim et al. [43] and Duan et al. [44] with slight modification. Briefly, 1.0 mM AAPH and 2.5 mM 2,2′-azobis-(2-amidinopropane) dihydrochloride were mixed with 1× phosphate-buffered saline and heated at 70 °C for 30 min. The ABTS solution was filtered using a 0.45-μm syringe filter and then adjusted to an absorbance of 0.65 ± 0.02 using 1× phosphate-buffered saline. After the addition of 980 µL of ABTS solution to 20 µL of the sample or standard, the absorbance was measured at 734 nm after 10 min of reaction.

### 4.6. Statistical Analysis

All data are expressed as mean and standard error for three replicates. Statistical analysis was performed using the SAS software (Enterprise Guide 7.1 version; SAS Institute Inc., Cary, NC, USA). Significant differences between treatment groups were evaluated at *p* < 0.05 using Tukey’s studentized test (HSD). Analysis of variance among experimental treatments were grouped using Tukey’s studentized test (HSD) at *p* < 0.05. The significant analysis between non-MeJA and MeJA and between non UV-B and UV-B treatments were performed by Student’s *t*-test. The relationship between the antioxidant activity and component contents under each treatment was analyzed using Pearson’s correlation coefficients.

## 5. Conclusions

This study investigated the antioxidant activity and antioxidant components of marigold flowers treated with the stress factors UV-B radiation and MeJA. Treatment with UV-B radiation and MeJA alone or in combination resulted in specific responses of marigold petal flavonoid content and antioxidant activity. Based on the morphological changes caused by these treatments, a UV-B radiation intensity of ≤1 W∙m^−2^∙s^−1^ and a MeJA concentration of ≤10 mM were reasonable treatment conditions within the experimental period. Under 1 W∙m^−2^∙s^−1^ UV-B irradiation, MeJA treatment resulted in an early increase in quercetin aglycone content and a later increase in glycoside content such as Q7G and Q3G. The antioxidant activity induced by UV-B irradiation and MeJA treatments exhibited a distinct difference in terms of ABTS radical scavenging activity. Single MeJA treatment accelerated the antioxidant activity, especially in early period. On the other hand, single UV-B irradiation decreased the activity in marigold petals. Interestingly, the decrease was relieved by MeJA treatment, in terms of compensating effect by MeJA. In overall conclusion, MeJA use has beneficial effect to increase of antioxidant components in marigold petals. The results suggest that the phytochemical content of marigold petals can be increased under the adequate control of environmental factors.

## Data Availability

Not applicable.
